# Psychological Risk Factors in the Transition from Suicidal Ideation to Suicidal Behavior in Young Adults

**DOI:** 10.3390/healthcare12181850

**Published:** 2024-09-14

**Authors:** Elif Yöyen, Merve Keleş

**Affiliations:** 1 Department of Psychology, Faculty of Humanities and Social Sciences, Sakarya University, Sakarya 54050, Turkey; 2 Department of Educational Psychology, College of Education, Texas Tech University, Lubbock, TX 79409, USA; mkeles@ttu.edu

**Keywords:** suicide, suicidal ideation, suicide attempt

## Abstract

**Background:** Suicidal behaviour, defined as acting with the intention of killing oneself and thinking about doing so, is a serious public health problem. Being able to list the risk factors in the process from suicidal ideation to suicidal behaviour is important in preventing suicide. **Objectives:** The study was conducted to examine the psychological variables that discriminate between individuals who attempt suicide and those who only have suicidal ideation. **Methods:** The sample of the study consisted of 108 individuals who attempted suicide and 197 individuals with suicidal ideation, aged 18–25 years. Data were collected using the Demographic Information Form, Anger/Impulsivity (A/I) and Hopelessness/Loneliness (H/L) subscales of the Suicide Probability Scale (SPS), Psychological Pain Scale (PPS), State and Trait Anxiety Scale (STAS), Beck Depression Inventory (BDI), Interpersonal Needs Questionnaire (INQ), Acquired Suicide Efficacy/Death Fearlessness Scale (ASE/DFS) and General Attitudes and Beliefs Scale Short Form (GABS-SF). **Results:** Results showed that anger/impulsivity (Wald = 4.827; *p* < 0.05), perceived burden on others (Wald = 8.613; *p* < 0.05), acquired suicide efficacy/death fearlessness (Wald = 13.377; *p* < 0.001), being female (Wald = 3.925; *p* < 0.05), presence of diagnosed psychiatric illness in the family (Wald = 5.705; *p* < 0.05), and receiving psychological support (Wald = 4.381. *p* < 0.05) variables are significant predictors of the transition from suicidal ideation to suicidal action. **Conclusions:** The identification of psychological factors between suicide attempters and suicide ideation groups may guide clinicians in the follow-up and treatment of individuals at risk of attempting suicide. In addition, the results may contribute to the development of new intervention, education and treatment programmes for suicide.

## 1. Introduction

Suicide (completed suicide) is a self-harming behaviour that results in death and is associated with the intention to die [1]. Someone dies every 40 s, and more than 700,000 people die by suicide every year. Between 2000 and 2019, the crude mortality rate fell by 29%, from 13.0 to 9.2 deaths per 100,000 population. In high-income countries, the decrease in suicide mortality was less than 5%. While the suicide rate increased by 28% in the United States, a 40% decrease was observed in Europe in the last 2 years, with the suicide rate falling from 21.9% in 2000 to 12.8% in 2019 [2]. In such a significant case, the scientific literature has identified some risk factors for suicide. The first risk factor is depression.

Depression, which is common throughout the world, is one of the oldest defined mental disorders. Severe depression can lead to suicide. In a study of psychiatric patients with major depressive disorder, Sokero et al. (2003) reported that 58 per cent of depressed patients had suicidal ideation during the current episode, and 15 per cent had attempted suicide during the initial period [3]. Another study found that depression, psychological distress and hopelessness were predictive of suicide risk [4]. 

Another risk factor is the concept of psychological pain. The concept of psychological pain (psychache), first used by Shneidman, is defined as hurt, suffering, pain, mental pain in the soul, and pain in the mind [5]. Montemarano et al. (2018), in their four-year longitudinal study examining the relationship between psychological pain and suicidal ideation, found a positive relationship between psychological pain and suicidal ideation and stated that there is a relationship between psychological pain changing over time and change in suicidal ideation [6]. In another study, Berardelli et al. (2019) investigated the impact of psychological pain on suicide risk and the relationship between psychological pain and psychiatric disorders. They reported that both psychological pain and the worst psychological pain experienced to date were associated with suicide risk. They recommended that psychological pain assessment should be routinely used in clinical practice to identify and treat suicide risk [7].

Another risk factor is the concepts of anger/impulsivity and hopelessness/loneliness. Duica, Dragulescu and Pirlog (2020) explained that impulsivity can be part of psychiatric disorders and that hopelessness and impulsivity are two indispensable components of suicidal behaviour [8]. Brezo et al. (2007) found in their study of young adults that individuals with suicide attempts had higher impulsivity scores than those who had suicidal thoughts but did not attempt suicide [9]. O’Connor and Sheehy (2000) suggested that the concept of hopelessness mediates the relationship between depression and suicidal behaviour [10]. Bagge et al. (2013) found that symptoms of depression and hopelessness were positively related to suicidal ideation and suicidal behaviour [11]. Chang et al. (2017) found that loneliness has a significant effect in predicting suicide risk and that there is a positive relationship between loneliness and self-harm behaviours [12]. 

The concepts of thwarted belonging and perceived burden on others are also risk concepts associated with suicide. According to Joiner’s (2005) Interpersonal Psychological Theory of Suicide (IPTS), suicidal ideation arises from a combination of thwarted belongingness and perceived burdensomeness [13]. Thwarted belongingness is the individual’s experience of alienation from others, not seeing themselves as part of family, friends or other groups that are important to them. In thwarted belongingness, the individual experiences loneliness and a lack of positive reciprocal relationships [14]. An example of loneliness is the thought that can be expressed as ‘I feel disconnected from other people today’; an example of a lack of positive relationships is the thought that can be expressed as ‘I have no one I can trust when I need them’. However, one of the basic human needs is to be part of a social structure, to form interpersonal relationships and to develop intimacy. Individuals want to belong to a group and to develop close relationships. The satisfaction of psychological needs after the fulfilment of physiological needs supports the individual to be healthy, happy and productive. When individuals feel they belong to a group, they feel safe and important. They can cope with stress and crisis with less damage by receiving support from their groups. Frequent and positive interaction with the groups to which the individual belongs, and the fact that the interaction is long-term and involves mutual love and approval, helps the individual to feel loved, valued and safe. In blocked belonging, the individual cannot feel these. 

Perceived burden to others is the individual’s perception of themselves as a burden to others. Others are people to whom the person is close. Perceived burden to others consists of two dimensions: self-burden and self-hatred. In the perceived burden dimension (unemployment, homelessness, imprisonment, physical illness, not being wanted (seeing oneself as an excess) and believing oneself to be a burden to the family), the individual’s perception of oneself as a burden to others is not always the result of cues gathered from around the individual but can sometimes develop as a result of unrealistic and inaccurate perceptions. The individual sees themselves as defective enough to be a burden to others [15]. The person who sees themselves as a burden may make statements such as ‘People would be better off if I left their lives’ and,—‘I make things difficult for everyone in my life’. As a result of the individual seeing themselves as a burden, thoughts of self-hatred dominate cognition. Self-hatred can manifest itself as low self-esteem, shame and self-blame. Self-hatred can be expressed in statements such as ‘I am useless’ and,—‘I hate myself’ [14].

Fatal or near-fatal suicide attempts are different from suicidal ideation. There are publications that state that the individual should also have acquired the capacity to commit suicide [15]. Another risk factor in this context is the concept of acquired suicidal competence. Van Orden et al. (2010) further defined the concept of acquired suicidal competence proposed by Joiner (2005) [13] arguing that suicidal behaviour occurs when an individual is repeatedly exposed to physically painful and/or frightening experiences [14]. When an individual is repeatedly exposed to physical pain and/or fear, the pain threshold increases and the fear of death decreases. This may reveal the individual’s lethal self-harm behaviour as a factor that increases suicidal competence. Childhood maltreatment, previous suicide attempts, exposure to suicidal behaviour, exposure to war, impulsivity, physical pain and fear-related processes may provide a direct pathway to suicide in acquired suicidal competence.

State and trait anxiety and irrational beliefs have also been reported as risk factors for suicide. Enatescu et al. (2020) investigated the relationship between suicidal ideation and state and trait anxiety in women in the prenatal period and found that anxiety had a predictive role for trait suicidal ideation [16]. Choi et al. (2011) investigated the relationship between anxiety symptoms and suicidal ideation in psychiatric patients [17]. The study found that anxiety symptoms and suicidal ideation were related. According to the results of a study investigating whether psychotherapy clients with and without suicidal ideation differ in terms of irrational beliefs, clients with suicidal ideation showed more irrational beliefs on the core dimension [18]. Another study reported that there was a significant relationship between suicidal ideation and irrational beliefs and that irrational beliefs predicted suicidal ideation [19].

Gender and genetics are also important concepts in suicide. The study by Stat-ham et al. (1998) reported that suicide attempts were more common in women than in men, but there was no difference in suicidal ideation between the sexes [20]. In addition, a review of the literature shows that there are studies showing that genetic factors and the presence of a diagnosed psychiatric illness in the family influence the risk of attempting suicide [21,22,23,24]. Studies conducted since the last century show that the presence of a mental disorder is an important risk factor for suicide deaths [25]. It is generally accepted that more than 90 per cent of those who die by suicide have a psychiatric diagnosis at the time of death [26]. In a study conducted to examine patterns of medication use prior to death in 1371 people who died by suicide, it was reported that 65.1% of the sample had been prescribed at least one medication, while 30.7% had been prescribed three or more medications. It was found that 51.7% of the sample were prescribed medication for a mental disorder [27]. 

As briefly outlined above in the light of the literature, several risk factors have been identified in studies of suicide, such as psychological distress, state and trait anxiety, hopelessness/loneliness, anger/impulsivity, depression, perception of being a burden to others, inhibition of belonging, acquired suicidal behaviour/no fear of death and irrational beliefs. Risk factors have been analysed as a single variable in some studies [16] and as a combination of two or more variables in others [4]. The aim of this study was to assess whether participants who attempted suicide differed from those who only had suicidal ideation in terms of the risk factors in the literature (psychological distress, state and trait anxiety, hopelessness/loneliness, anger/impulsivity, depression, perception of being a burden to others, inhibition of belonging, acquired suicidal act/fear of death and irrational beliefs) and then to examine which psychological factors were significant predictors in the correct classification of suicide attempt and suicidal ideation. There are no studies in the literature that have investigated which psychological factors are significant predictors of the correct classification of suicide attempts and suicidal ideation. The innovative aspect of this study is that it aims to fill this gap in the literature. Correct classification of risk factors in the transition from suicidal ideation to suicidal behaviour may play a preventive role in educational, psychological and health policy decisions. It may guide clinicians in the organisation of psychoeducation and the identification of suicide risk. 

## 2. Materials and Methods

The study was approved by the Ethics Committee in Turkey with a decision dated 18 February 2021 and numbered 57. Informed consent was obtained from participants and no identity information was requested. The independent variables examined in logistic regression in the study using the relational screening model were anger/impulsivity, perceived burden on others, acquired suicidal efficacy/fear of death, depression, psychological pain, gender, receiving psychological support, diagnosed psychiatric illness, psychiatric diagnosis in the family and psychiatric drug use. The dependent variable is the categories of attempted suicide or suicidal ideation, which express the status in relation to suicide. In the study, the suicidal ideation group and the suicide attempt group were determined as follows: Question 9 of the Beck Depression Inventory was used to determine people with suicidal ideation. This question consists of the following options: (a) I do not think about killing myself, (b) I sometimes think about killing myself but I cannot do it, (c) I would like to kill myself very much and, (d) If I find an opportunity I will kill myself. Except for those who answered ‘(a) I have no thoughts of killing myself’, those who ticked the other options were classified as having suicidal thoughts. Suicide attempt is the group of participants who answered yes to the question ‘Have you ever tried to kill yourself? The sample size of this study was determined by considering the number of samples required for different population sizes (*n* = 384) [28].

### 2.1. Participants

The study sample consisted of 305 adults aged 18–25 years. The study included 108 people who had attempted suicide and 197 people with suicidal ideation. As the participants who had attempted suicide and those with suicidal ideation were relatively difficult to reach and unknown, snowball sampling was used. Snowball sampling is a sampling method that is generally used when people with the desired characteristics are rare or unknown in society [29]. 

Participants were included in the study if they agreed to participate and gave informed consent as a prerequisite for study participation. Exclusion criteria included general medical conditions, neurodevelopmental disorders, being under the influence of drugs and/or alcohol, being under 18 years of age and not being over 25 years of age. 

Data were collected online via Google Forms between 19 February 2021 and 1 August 2021. The informed consent form and other measures were made available online and communicated to participants via social media communication channels (Facebook, Instagram, Twitter, etc.). The consent form includes information about the purpose of the research, the expected duration, the conditions of participation and the principles of confidentiality. Participation is voluntary and no identifying information will be collected from participants. The researchers’ contact details were given at the end of the form for those participants who wished to receive psychological support. 

### 2.2. Analysis

In the study in which 313 participants were included, the data of 8 participants were excluded from the analysis due to outliers. After the data of 8 participants were excluded from the analyses, the data of 305 participants, 108 of whom attempted suicide and 197 of whom had only suicidal ideation, were evaluated. Logistic regression analysis (backward stepwise) was used to determine the discriminating factors between suicide attempt and suicidal ideation. Binary logistic regression analysis was used to determine the intervening variables in the transition from suicidal ideation to suicide attempt. Binary logistic regression analysis is a type of logistic regression analysis used when the dependent variable is a qualitative variable with two categories. In order to obtain reliable results in this analysis, some assumptions and requirements must be met. According to the number of predictor variables, there should not be too few individuals in the groups considered, there should not be multicollinearity between the predictor variables and there should not be extreme values. Logistic regression analyses were performed on 108 people with suicide attempts and 197 people with suicidal ideation, so the requirement of not having too few individuals in the groups was met. The tolerance value (TV) and variance increase factor (VIF) values were analysed to avoid the problem of multicollinearity. Tolerance values greater than 0.20 and VIF values less than 10 mean that there is no multicollinearity problem. In the analyses, it was observed that all tolerance values were greater than 0.20 and VIF values were less than 10. Accordingly, it can be said that there is no multicollinearity problem among the predictor variables. Outlier analyses were completed prior to the start of the analyses. As a result, it can be said that all assumptions required for binary logistic regression analysis are met. All independent categorical variables (gender, receiving psychological support, diagnosed psychiatric illness, psychiatric diagnosis in the family and, use of psychiatric medication) and all continuous variables (anger/impulsivity, perceived burden on others, acquired suicidal competence/fear of death, depression and, psychological pain) that were significant only in univariate analyses in relation to suicide attempt and suicidal ideation were analysed using the backward stepwise (conditional) method of binary logistic regression analysis.

### 2.3. Data Collection Tools

Demographic information form: The form prepared by the researchers includes personal information such as age, gender, diagnosed psychiatric illness, psychotropic medication use, psychiatric diagnosis in the family and, psychological support. This information and the items of the scales used in the study, explained below, are included in the Supplementary File S1. Data was collected with these items.

Suicide Probability Scale (SPS): It is a self-report scale developed by Cull and Gill in 1988 to assess suicide risk in adolescents and adults. The scale, whose validity and reliability study was conducted by Batıgün and Hisli in 2018, has four sub-dimensions: Social Support/Self-Perception, Anger/Impulsivity, Hopelessness/Loneliness, and Suicidal Thoughts [30]. In this study, the Hopelessness/Loneliness and Anger/Impulsivity subscales were included in the analyses, and the Cronbach’s alpha value was found to be 0.77 for the Anger/Impulsivity subscale and 0.77 for the Hopelessness/Loneliness subscale and 0.93 for all scale items.

Psychological Pain Scale (PPS): This is a 13-item self-report scale based on Shneidman’s definition of chronic, free-floating, non-situation-specific psychological pain resulting from the failure to meet vital psychological needs [5]. Responses to questions on this five-point Likert scale range from never to always, or from strongly disagree to strongly agree. It was developed to investigate the relationship between psychological pain and suicidal ideation [31]. In the reliability analysis conducted in this study, the Cronbach’s alpha value was found to be 0.97, which indicates the frequency of psychological pain rather than its severity.

State-Trait Anxiety Scale (STAS): Developed by Spielberger et al. (1971), the STAS consists of two separate scales with a total of forty items. The State Anxiety Inventory is a measure of how a person feels at a given moment, while the Trait Anxiety Inventory is a measure of how a person feels in general [32]. In this study a Cronbach alpha value of 0.93 was calculated for the State Anxiety Inventory, 0.91 for the Trait Anxiety Inventory and 0.95 for the total scale.

Beck Depression Inventory (BDI): Developed by Aaron T. Beck in 1961 to assess depression, the BDI is a self-report scale designed to objectively measure an individual’s symptoms of depression. It consists of 21 questions that assess cognitive, emotional and somatic symptoms of depression, and the answers to these questions provide information about the severity of depression. [33]. The Cronbach alpha value of the scale, which aims to determine the degree of depression [34], was found to be 0.93 in this study.

The Interpersonal Needs Questionnaire (INQ): The scale was developed to measure the perceived burden on others and thwarted sense of belonging, with five items measuring perceived burden on others and the other five items measuring thwarted sense of belonging, and consists of ten items in total. As the score obtained from the scale increases, it is assumed that the individual’s interpersonal needs also increase [35]. In this study, the Cronbach’s alpha value was calculated as 0.91 for the total scale, 0.93 for the perceived burden on others factor and 0.88 for the thwarted sense of belonging factor.

Acquired Suicide Efficacy/Death Fearlessness Questionnaire (ASE/DFQ): This is a seven-item self-report scale developed by Ribeiro et al. (2014) to determine the level of acquired suicide competence. Higher scores in a single-factor structure of the seven-point Likert-type scale indicate greater fearlessness about death [36]. In the reliability analysis conducted in this study, the Cronbach alpha value was found to be 0.86. 

General Attitudes and Beliefs Scale Short Form (GABS-SF): This scale was developed by Lindler, Kirkby, Wertheim and Birch (1999) to measure irrational beliefs in adults and adolescents. The items of the scale, which consists of 26 items in total, are answered on a five-point Likert scale. Thus, each question has a value between 1 and 5. The scale consists of 7 factors: rationality, self-devaluation, irrational beliefs of success, irrational beliefs of approval, search for comfort, expectation of justice and devaluation of others. As all subscales except rationality express irrational beliefs, this subscale was reverse coded in the analysis studies [37]. The reliability of the scale for this study was determined to be 0.92.

## 3. Results

The demographic characteristics of the participants are shown in Table 1. When the suicidal behaviour status of the participants was assessed, 108 (35.40%) had attempted suicide and 197 (64.59%) had suicidal ideation only. The characteristics of the participants with suicide attempts were as follows: 55 (50.9%) were female and 53 (49.1%) were male. A total of 56 (51.9%) of the participants did not have a history of diagnosed psychiatric illness, while 52 (48.1%) did; 75 (69.4%) were receiving psychiatric medication, 33 (30.6%) were not; 57 (52.8%) of the participants had no family member with a psychiatric diagnosis, while 51 (47.2%) had a family member with a psychiatric diagnosis, and 84 (77.8%) of the participants did not receive psychological support, while 24 (22.2%) did. Of the participants with suicidal ideation, 71 (36%) were women and 126 (64%) were men. Of the participants, 135 (68.5%) did not have a history of psychiatric illness and 62 (31.5%) did; 161 (81.7%) participants were not taking any psychiatric medication, while 36 (18.3%) were; 135 (68.5%) of the participants had no other family member with a psychiatric diagnosis, while 62 (31.5%) of the participants had a family member with a history of a diagnosed psychiatric illness; and 173 (87.8%) of the participants did not receive psychological support, while 24 (12.2%) did. 

The mean hopelessness/loneliness score of the participants with suicide ideation was 20.315 (SD = 4.141), the mean anger/impulsivity score was 12.787 (SD = 3.748), the mean interpersonal needs score was 38.741 (SD = 12.038), the mean burdening others score was 13.208 (SD = 7.653), the mean thwarted belongingness score was 25.533 (SD = 7.090), the mean suicidal competence/fearlessness of death score was 33.731 (SD = 11.058), the mean depression score was 26.756 (SD = 11.095), the mean psychological distress score was 45.020 (SD = 14.581), the mean state anxiety score was 51.178 (SD = 10.492), the mean trait anxiety score was 55.563 (SD = 9.480) and the mean general attitudes and beliefs score was 84.909 (SD = 17.216).

The mean hopelessness/loneliness score of the suicide attempt participants was 20.898 (SD = 3.843),the mean anger/impulsivity score was 14.750 (SD = 4.326), the mean interpersonal needs score was 43.676 (SD = 13.853), the mean burdening others score was 17.444 (SD = 8.999), the mean thwarted belongingness score was 26.231 (SD = 6. 917), suicide competence/fearlessness of death score mean 37.907 (SD = 10.131), the mean depression score was 31.444 (SD = 12.490), the mean psychological pain score was 49.991 (SD = 13.736), the mean state anxiety score was 53.065 (SD = 11.264), the mean trait anxiety score was 57.529 (SD = 10.744) and the mean general attitudes and beliefs score was 89.000 (SD = 20.192). The results of the descriptive statistics of the scales are presented in Table 2.

In this study, the risk factors that emerged from the literature review to determine the factors that differentiate suicide attempt and suicide ideation are as follows: anger/impulsivity, perceived burden on others, acquired suicidal efficacy/fear of death, depression, psychological pain, gender, receiving psychological support, diagnosed psychiatric illness, psychiatric diagnosis in the family and, use of psychiatric medication. These factors were analysed using the backward stepwise (conditional) method of binary logistic regression analysis. The results are shown in Table 3. The variables in the study were analysed in five stages using backward stepwise logistic regression analysis. Anger/impulsivity (Wald = 4.827; *p* < 0.05), perceived burden on others (Wald = 8.613; *p* < 0.05), acquired suicidal efficacy/fearlessness of death (Wald = 13. 377; *p* < 0.001), gender (Wald = 3.925; *p* < 0.05), have psychiatric diagnosis in the family (Wald = 5.705; *p* < 0.05) and, receiving psychological support (Wald = 4.381. *p* < 0.05) were found statistically significant in the regression model ($X_{\left(6\right)}^{2}$ = 52.850; *p* < 0.001).

According to the results of the study, the risk of attempting suicide among individuals with suicidal ideation was found to be 1.081 times (95% CI = 1.008–1.159) with one unit increase in anger/impulsivity score, 1.051 times (95% CI = 1.017–1. 086) with one unit increase in acquired suicidal efficacy/fear of death score, and 1.050 times (95% CI = 1.023–1.078), female gender by 1.697 times (95% CI = 1.006–2.862), having a diagnosed psychiatric illness in the family by 1.901 times (95% CI = 1.122–3.220) and receiving psychological support by 2.090 times (95% CI = 1.048–4.168).

The Nagelkerke $R^{2}$ coefficient showed that the model significantly predicted 22% of the variation between those with suicidal ideation only and those with suicide attempts. In general, the model correctly classified 71.5% of participants as having attempted suicide and having suicidal ideation. The results of the model’s prediction of the proportion of individuals with suicide attempts and suicidal ideation are shown in Table 4. As shown in Table 4, the sensitivity obtained in step 5 of the multivariate logistic regression analysis was 87.8% and the selectivity was 41.7%. The overall correct classification percentage was 71.5%.

## 4. Discussion

As a result of the study, it was found that the participants who attempted suicide scored higher than the participants who had suicidal ideation on the scales of hopelessness/loneliness, anger/impulsivity, interpersonal needs, perceived burden on others, hindered belonging, acquired suicidal activity/fearlessness of death, depression, psychological pain, state and trait, general attitude and belief. In the study, the ratio of all independent variables explaining the dependent variable was determined as 0.219 (Nagelkerke $R^{2}$ = 0.219). In addition, in the logistic regression analysis, the following factors were ranked in terms of their power to influence the transition from suicidal ideation to suicide behaviour: receiving psychological support, having a psychiatric disorder in the family, being a female, anger/impulsivity, being a perceived as a burden to others and the acquired suicide efficacy. The results of the study are consistent with the literature.

There is evidence in the literature that hopelessness and loneliness are associated with suicidal ideation and suicide attempts [38,39]. There are studies showing that hopelessness significantly and positively predicts suicidal ideation [12,40]. There are several studies showing a relationship between anger and impulsivity and suicide risk [41,42]. In a study examining anger, impulsivity and suicide risk, anger and impulsivity were found to be associated with suicide risk, and impulsivity was reported to be higher in the suicidal group than in the non-suicidal and healthy groups [41]. In addition to studies showing that there is a relationship between anger and suicide attempts and that suicidal behaviour is related to impulsivity [43], there are studies examining the effects of the concepts of frustration, perceived burden on others and acquired suicidal competence on suicidal ideation and attempts by evaluating the theory of interpersonal psychological needs. As a result of this review, they reported that perceived burden on others had a stronger relationship with suicidal ideation than thwarted belongingness. In the same review, three of the seven studies that examined the interaction effects between suicide attempt and perceived burden on others, thwarted belongingness and acquired suicidal efficacy were found to be significant, while four were found to be nonsignificant [44]. However, this study was criticised by Chu et al. (2017) for not conducting a meta-analysis and for examining a limited number of databases [15]. Chu et al. (2017), in their meta-analysis study examining the components of interpersonal suicide theory, concluded that the interaction between frustrated belongingness and perceived burden on others was associated with suicidal ideation and that the interaction between frustrated belongingness, perceived burden on others and acquired suicidal efficacy was significantly associated with past suicide attempts [15]. A review of the literature shows that many studies have reported a strong association between depression and suicidal behaviour [45,46,47]. Conejero et al. (2018), in a review study examining the relationship between psychological and physical pain with depression and suicidal behaviour, stated that psychological pain was an important dimension of depressive disorders. They also found that psychological pain was associated with a higher risk of suicidal ideation and suicidal behaviour [48]. A meta-analysis of the relationship between suicidal ideation and attempts and psychological pain compared 20 studies of people with or without current or lifetime suicidal ideation or with or without a history of suicide attempts. The study found that high levels of psychological pain were associated with suicidal ideation and suicide attempts [49]. There are studies reporting that state and trait anxiety are associated with suicide risk [50,51]. Studies investigating the relationship between irrational beliefs and suicidal ideation reported that there was a significant relationship between the two variables and that irrational beliefs predicted suicidal ideation [52,53]. All of these findings are risk factors associated with suicide.

The second finding of the study was that the anger/impulsivity, perceived burden on others, acquired suicidal efficacy/fear of death, being female, having a diagnosed psychiatric illness in the family and receiving psychological support variables were significant predictors in the analyses conducted to determine the possible predictors distinguishing those who had suicidal ideation from those who had attempted suicide. The results of the study are in line with the literature.

According to the results of the study, anger/impulsivity was found to be a variable that significantly discriminated between suicide attempt and suicidal ideation. To date, several studies have shown that anger and impulsivity are associated with suicide risk [54,55]. Dillon, Van Voorhees, and Elbogen (2019) concluded that anger significantly predicted both suicidal ideation and suicide attempts in their longitudinal study of 34,653 individuals [19]. At the same time, high impulsivity is thought to facilitate the transition from suicidal ideation to suicidal behaviour [56,57]. In light of the studies in the literature, anger and impulsivity can be described as a trait that may predispose individuals to suicidal and violent behaviour. The fact that anger and impulsivity are important in the transition from suicidal ideation to suicide attempt can be explained by the fact that anger/impulsivity is associated with violent behaviour. The fact that anger/impulsivity facilitates the transition from suicidal ideation to suicidal behaviour may be related to the nature of impulsivity. Acting without thinking, inability to plan, acting suddenly without preparation (motor activation) and behaviours that result in rapid and/or incorrect performance in difficult situations are the nature of anger/impulsivity. The person often exhibits behaviours such as acting without thinking, making quick cognitive decisions, focusing on the present or not thinking about the future. The person’s reactions take the form of a tendency to react quickly and unplanned to internal or external stimuli, without considering the negative consequences for themselves or others. With these characteristics, it is not possible for the person to re-evaluate his/her suicidal thoughts (thinking about the problems he/she thinks are driving him/her to suicide, seeking alternative solutions to these problems or, persevering to solve the problems).

According to the research findings, perceived burden on others and acquired suicidal efficacy/fearlessness of death were found to be significant predictors in correctly classifying individuals with suicide attempts and individuals with suicidal ideation only. In Joiner’s (2005) interpersonal psychological theory of suicide [58], perceived burden on others and thwarted belongingness are explained as necessary factors for the development of suicidal ideation. In our study, perceived burden on others was found to be a significant variable in the transition from suicidal ideation to suicide attempt. In their systematic review of 58 articles from 66 different studies, Ma et al. (2016) reported that perceived burden on others had a stronger relationship with suicidal ideation than thwarted belongingness [44]. The role of perceived burden on others, thwarted belongingness and acquired suicidal efficacy in predicting future suicide attempts was examined in a group of inpatients in a psychiatric hospital, and participants with suicidal ideation and suicide attempts were followed up for 12 months. The results showed that the interaction effect of perceived burden on others, thwarted belongingness and acquired suicidal efficacy did not predict future suicide attempts but that perceived burden on others had a significant main effect [59]. The finding of acquired suicidal efficacy as a significant factor in the transition from suicidal ideation to suicide attempt supports Joiner’s (2005) interpersonal psychological needs theory [13]. Joiner (2005) suggested that fatal or near-fatal suicide attempts are most likely to be associated with inhibited belongingness, perceived burden on others (and hopelessness about both), decreased fear of death and increased tolerance of physical pain [13,14]. In line with the findings of this study, it is suggested that fear of death should be considered in the assessment of suicide attempts. The perceived burden on others and the acquired suicide competence/fear of death can be explained by the following mechanism in the process from suicidal ideation to suicide attempt. One of a person’s most basic psychological needs is the need to belong. A person communicates and interacts with other people in their social relationships from birth to death. Individuals who have a group, a social environment and an environment full of people who love and support them feel that they belong to the groups they are a part of and that meet their needs for love, security, feeling valuable and special and recognition, but if they cannot have this, if they are alienated from their environment and if their social ties are damaged, the individual does not feel that they belong to any place, person, institution or phenomenon. An individual who cannot maintain their relationship with others with social ties has difficulty coping with life stressors and believes that they cannot find people to help them in stressful events. This is caused by the individual believing that they are a burden to others (people in their social environment to whom they do not feel they belong but who are still around them) and seeing themselves as incomplete, flawed, defective, useless and losing their self-esteem. It can lead to feelings of self-hatred, guilt and shame. It can lead to a sense of hopelessness that the individual is a burden to others because the individual’s sense of belonging is blocked. Hopelessness can lead the individual into a depressive mood, and if the fear of death is reduced in addition to suicidal thoughts, the motivation for suicide can become stronger.

Gender, family history of psychiatric illness and receipt of psychological support were found to be significant predictors of the transition from suicidal ideation to suicide attempt. The study by Statham et al. (1998), which reported lifetime rates of suicidal ideation and behaviour, reported that suicide attempts were more common in women than in men, but there was no difference in suicidal ideation between the sexes [20]. Bertolote and Fleischmann (2002), in an international study of WHO member countries, reported that male suicide rates in countries other than China were more than three times higher than female rates in 1950 and almost five times higher in 1995 [26]. Moscicki (1994) reported that suicide attempts were more common among women and completed suicides were more common among men. This gender-specific difference may be explained by the use of different suicide methods depending on gender. As men choose more lethal methods of suicide, their mortality rates may be higher. Another explanation is that the lower rate of completed suicide in women may be explained by the fact that they have higher rates of depression and higher rates of treatment than men [60].

There are studies showing that genetic factors and the presence of a diagnosed psychiatric illness in the family influence the risk of attempting suicide [21,23]. Kendler et al. (2020), who reviewed the source of intergenerational transmission (from parent to child) in suicide attempts and deaths by suicide, concluded that genetic factors and parenting are moderately strong and equally effective in the intergenerational transmission of suicide attempts [21]. It has been reported that two-thirds of people with serious suicide attempts reported a history of maternal depression, and one-third reported a history of paternal depression and alcoholism [20]. Roy et al. (1991) found that genetic factors associated with suicide largely represent a genetic predisposition to suicide-related psychiatric disorders [61]. The results indicate that the presence of a diagnosed psychiatric disorder in the family may be an important risk factor in the transition from suicidal ideation to suicide attempt. The results of our study are consistent with the information in the literature.

According to the results of the study, the receipt of psychological support was found to be a significant predictor in correctly classifying people with suicide attempts and people with suicidal ideation only. Although there have been many studies of risk factors for suicide, it is known that clinicians are rarely better than chance at assessing suicide risk [62]. There is evidence that the risk of suicide increases in people with a history of mental illness and psychiatric hospitalisation [63]. One study reported that the most common reason for undergraduates concealing suicidal thoughts from psychiatrists was fear of hospitalisation, while the most common reason for concealing them from psychologists or therapists was embarrassment. Other reasons included being judged and not taken seriously [20]. The reasons why receiving psychological support is an important risk factor for the transition from suicidal ideation to suicide attempt can be listed as the fact that these individuals have more psychiatric diagnoses, mental health professionals avoid asking clients about their suicidal ideation, clients tend to hide their suicidal ideation because they are afraid of being hospitalised, judged or stigmatised and the inability to clarify the boundaries of confidentiality during the therapeutic process and the inability to build trust in therapy. Failure to clarify the boundaries of confidentiality and to build trust in therapy may be related to the client’s inability to open up and share suicidal thoughts. In addition, the process may lead to suicide attempts because clinicians fail to identify people with suicidal thoughts, or even if they do, the correct intervention is not applied. It is thought that reducing the stigma associated with reporting and experiencing suicidal thoughts, and discussing suicide openly and directly, may support suicide prevention efforts.

## 5. Conclusions

The importance of this study is that it analyses together psychological variables that are risk factors in the transition from suicidal ideation to suicidal action. Risk factors that have been analysed individually or as two or more variables in previous studies have been evaluated as a whole and together in this study. This is the innovative aspect of the study. When the results of the study were evaluated, it was found that anger/impulsivity, perception of being a burden to others, acquired suicidal activity/fear of death, being female, having a diagnosed psychiatric illness in the family and receiving psychological support may be risk factors in the transition from suicidal ideation to suicide attempt. In this context, it is recommended that mental health professionals take these factors into account when assessing suicide risk, questioning individuals more about their suicidal thoughts and plans and developing an action plan accordingly. In addition, this was a cross-sectional study. In future studies, it is recommended that risk factors that help distinguish the transition from suicidal thoughts to suicide attempts should be investigated in longitudinal studies.

### 5.1. Limitations

The results of this study should be interpreted in light of its limitations. As this is a cross-sectional study, it is limited in that no causal conclusions can be drawn from the results. Cross-sectional studies cannot establish causality because of the lack of temporal order. They can define associations but not cause effect relationships. Longitudinal studies, on the other hand, have a greater ability to establish causal relationships because they can assess the temporal order of events. Therefore, longitudinal studies are recommended for future research.

Another limitation is the limited sample size due to the difficulty of reaching people who have attempted suicide in the general population. Conducting a longitudinal study with larger sample groups may be more generalisable and may reveal the causal relationship. In addition, the fact that the data in this study were collected online (due to the pandemic) is another limitation that needs to be addressed. Face-to-face data collection is recommended for future research.

Another limitation of this study is that the data were collected during the pandemic period. There are studies on the negative effects of the pandemic process on the mental health of all people, especially young people [64,65,66,67]. In this context, the data have the limitation that they may reflect the negative mental health effects of the pandemic process. It is recommended that future studies take this limitation into account.

### 5.2. Recommendations for Future Researchers and Public Health Policy Makers

Young adults were included in this study. Similar studies can be conducted with sample groups such as children and elderly people who are at risk for suicide.

In this study, hopelessness/loneliness was analysed as a single dimension. In future studies, it is suggested that different structures of hopelessness, such as state and trait hopelessness, should be addressed, similarly, loneliness should be addressed as state and trait loneliness or subjective and objective loneliness.

In this study, anger/impulsivity was handled as a single dimension. In future studies, it is suggested that anger should be handled with different structures, such as inward anger and outward anger.

For the risk factors identified in this study, group therapy and psychoeducation programmes can be prepared to improve the coping and problem solving skills of individuals. 

By increasing the number of community mental health centres and the number of competent psychologists working in these centres, it can be ensured that everyone who needs psychological support can receive it in a short time. Thus, people who may be in the suicide risk group can be reached faster and necessary measures can be taken.

The number of applied courses, including suicide risk assessment and suicide prevention programmes, can be increased in psychology and clinical psychology programmes of universities.

All psychologists employed in the field can be regularly trained in crisis management and suicide risk assessment.

Suicide prevention centres can be established and expanded. These centres can be organised to provide 24/7 active service by psychologists who are experts in the field of suicide risk assessment.

A suicide prevention hotline (via telephone and online communication tools) can be established and organised in such a way that it can be accessed at any time by individuals seeking help, and that suicide risk assessment and crisis management can be provided by expert psychologists.

## Figures and Tables

**Table 1:** Demographic characteristics of the participants.

Variables	Sub-Variables	Suicide Attempters (*n* = 108)	Suicidal Ideators (*n* = 197)
Age	Mean ± SD	27.42 ± 7.5	26.07 ± 7.5
	Min.–Max.	18–20	18–25
		Frequency (*n*)—Percentage (%)	Frequency (*n*)— Percentage (%)
Gender	Female	55 (50.9%)	71 (36%)
	Male	53 (49.1%)	126 (64%)
Diagnosed psychiatric illness	No	56 (51.9%)	135 (68.5%)
	Yes	52 (48.1%)	62 (31.5)%
Psychiatric drug use	No	75 (69.4%)	161 (81.7%)
	Yes	33 (30.6%)	36 (18.3%)
Psychiatric diagnosis in the family	No	57 (52.8%)	135 (68.5)%
	Yes	51 (47.2%)	62 (31.5%)
Psychological support	No	84 (77.8%)	173 (87.8%)
	Yes	24 (22.2%)	24 (12.2%)

**Table 2:** Descriptive statistics of scale scores according to participants’ suicide status.

Scale/Subscale	Suicide Status	*n*	$\bar{X}$	SD
Hopelessness/Loneliness Subscale	Suicidal ideators	197	20.315	4.141
	Suicide attempters	108	20.898	3.843
Anger/Impulsivity Subscale	Suicidal ideators	197	12.787	3.748
	Suicide attempters	108	14.750	4.326
Interpersonal Needs Questionnaire Scale	Suicidal ideators	197	38.741	12.038
	Suicide attempters	108	43.676	13.853
Perceived Burden on Others Subscale	Suicidal ideators	197	13.208	7.653
	Suicide attempters	108	17.444	8.999
Thwarted Belonging Subscale	Suicidal ideators	197	25.533	7.090
	Suicide attempters	108	26.231	6.917
Acquired Suicide Efficacy/Fearlessness of Death Scale	Suicidal ideators	197	33.731	11.058
	Suicide attempters	108	37.907	10.131
Beck Depression Inventory	Suicidal ideators	197	26.756	11.095
	Suicide attempters	108	31.444	12.490
Psychological Pain Scale	Suicidal ideators	197	45.020	14.581
	Suicide attempters	108	49.991	13.736
State Anxiety Scale	Suicidal ideators	197	51.178	10.492
	Suicide attempters	108	53.065	11.264
Trait Anxiety Scale	Suicidal ideators	197	55.563	9.480
	Suicide attempters	108	57.529	10.744
General Attitudes and Beliefs Scale	Suicidal ideators	197	84.909	17.216
	Suicide attempters	108	89.000	20.192

**Table 3:** Logistic regression analysis for determination of factors distinguishing suicide attempt and suicidal ideation (backward stepwise, step 5).

Step 5 (Final Model)							
Variables	β	s_β_	Wald	*p*	Exp(β)	95% Confidence İnterval (CI) for Exp(B)	
						Lower	Upper
Anger/Impulsivity	0.078	0.035	4.827	0.028	1.081	1.008	1.159
Perceived Burden on Others	0.050	0.017	8.613	0.003	1.051	1.017	1.086
Acquired Suicide Efficacy/Fearlessness of Death	0.049	0.013	13.377	0.000	1.050	1.023	1.078
Gender (female)	0.529	0.267	3.925	0.048	1.697	1.006	2.862
Psychiatric diagnosis in family (Yes)	0.642	0.269	5.705	0.017	1.901	1.122	3.220
Receiving psychological support (Yes)	0.737	0.352	4.381	0.036	2.090	1.048	4.168
Constant	−4.778	0.753	40.248	0.000	0.008		

Nagelkerke $R^{2}$ = 0.219; Omnibus $X_{\left(6\right)}^{2}$ = 52.850 (*p* < *0*.001); Hosmer ve Lemeshow = *p* = 0.812 (*p* > 0.05); β: Regression coefficient; s_β_: Standardized Regression Coefficient; Exp(β): Expression Regression Coefficient.

**Table 4:** Classification table obtained as a result of multivariate logistic regression analysis.

	Observed	Guess		Percentage of Correct Classification	
		Suicidal Ideators	Suicide Attempters		
Step 5	Suicidal ideators	173	24	87.8	Sensitivity
	Suicide attempters	63	45	41.7	Selectivity
Overall Correct Classification Percentage				71.5	Accuracy

## Data Availability

The data presented in this study are available to all researchers.

## Supplementary Materials

The following supporting information can be downloaded at: https://www.mdpi.com/article/10.3390/healthcare12181850/s1, Supplementary File S1: Data Collection Tools.
