# Supplementary material for: Psychological Risk Factors in the Transition from Suicidal Ideation to Suicidal Behavior in Young Adults
Source: Healthcare (Basel). 2024 Sep 14;12(18):1850. doi: 10.3390/healthcare12181850 (PMC11431594; doi:10.3390/healthcare12181850)
Supplement: Supplementary file 1 [file healthcare-12-01850-s001.zip › healthcare-3142741-supplementary.pdf]

Supplementary File S1: Data Collection Tools

ANNEX1: Suicide Probability Scale (SPS)

	**Degree of Defining You**			
	**% 0**	**% 30**	**%70**	**%100**
**Anger/Impulsivity**				
**1.** I throw things when I get angry.				
2. I tend to make sudden decisions or do things without thinking.				
**3.** I think of things that are too bad to tell others.				
**4.** I have hostile feelings towards others.				
**5.** I think that people have hostile feelings towards me.				
**6.** I break things when I get angry.				
**Hopelessness/Loneliness**				
**7.** I feel isolated from people.				
**8.** I feel unbearably lonely.				
**9.** I feel that I could make many changes in my life if I could start all over again.				
**10.** I find it difficult to maintain friendships with people I like.				
**11.** I don't think things will ever get better.				
**12.** I worry about money.				
**13.** I feel tired and have no interest in anything.				

ANNEX2: Psychological Pain Scale (PSS)

1. I feel psychological pain

☐ 1 (Never) ☐ 2 (Somitemes) ☐ 3 (Often) ☐ 4 (Very Often) ☐ 5 (Always)

2. I feel the pain inside me

☐ 1 (Never) ☐ 2 (Somitemes) ☐ 3 (Often) ☐ 4 (Very Often) ☐ 5 (Always)

3. My psychological pain hurts me more than any physical pain.

☐ 1 (Never) ☐ 2 (Somitemes) ☐ 3 (Often) ☐ 4 (Very Often) ☐ 5 (Always)

4. My pain makes me want to scream

☐ 1 (Never) ☐ 2 (Somitemes) ☐ 3 (Often) ☐ 4 (Very Often) ☐ 5 (Always)

5. My pain makes my life look pitch black

☐ 1 (Never) ☐ 2 (Somitemes) ☐ 3 (Often) ☐ 4 (Very Often) ☐ 5 (Always)

6. I can't understand why I'm in pain

☐ 1 (Never) ☐ 2 (Somitemes) ☐ 3 (Often) ☐ 4 (Very Often) ☐ 5 (Always)

7. I feel terrible psychologically.

☐ 1 (Never) ☐ 2 (Somitemes) ☐ 3 (Often) ☐ 4 (Very Often) ☐ 5 (Always)

8. It hurts because I feel empty inside.

☐ 1 (Never) ☐ 2 (Somitemes) ☐ 3 (Often) ☐ 4 (Very Often) ☐ 5 (Always)

9. My soul hurts

☐ 1 (Never) ☐ 2 (Somitemes) ☐ 3 (Often) ☐ 4 (Very Often) ☐ 5 (Always)

Please continue filling out the scale by answering the following questions as indicated.

1. Strongly disagree 2. Disagree 3. Not sure 4. Agree 5. Strongly agree

10. I can't stand the pain anymore

☐ 1 (Strongly disagree) ☐ 2 (Disagree) ☐ 3(Not sure) ☐ 4(Agree) ☐ 5 (Strongly agree)

11. Because of my pain, I am in an unbearable situation

☐ 1 (Strongly disagree) ☐ 2 (Disagree) ☐ 3(Not sure) ☐ 4(Agree) ☐ 5 (Strongly agree)

12. I am torn apart by my pain

☐ 1 (Strongly disagree) ☐ 2 (Disagree) ☐ 3(Not sure) ☐ 4(Agree) ☐ 5 (Strongly agree)

13. My psychological pain affects everything I do.

☐ 1 (Strongly disagree) ☐ 2 (Disagree) ☐ 3(Not sure) ☐ 4(Agree) ☐ 5 (Strongly agree)

ANNEX3: State and Trait Anxiety Scale (STAS)

.

		NOT AT ALL	A LITTLE	MUCH	ENTIRELY
1	I am calm right now				
2	I feel safe				
3	My nerves are on edge right now				
4	I feel regretful right now				
5	I am at peace right now				
6	I am not feeling well at all right now				
7	I am worried about what will happen to me				
8	I feel rested right now				
9	I am anxious right now				
10	I feel relaxed				
11	I have self-confidence right now				
12	I am irritable right now				
13	I feel my nerves are on edge right now				
14	I feel relaxed				
15	I am happy right now				
16	I am anxious right now				
17	I feel overwhelmed with excitement right now				
18	I am happy right now				
19	I am in a good mood right now				
20	I am calm right now				

		Almost never Sometimes	Sometimes	A lot of the time	Almost always
21	I am usually in a good mood				
22	I usually get tired easily				

23	I usually cry easily				
24	I want to be as happy as others				
25	I miss opportunities because I can't make quick decisions				
26	I feel rested				
27	I am usually calm, collected and cool-headed				
28	I feel that difficulties are piling up so much that I can't overcome them				
29	I worry about unimportant things				
30	I am usually happy				
31	I take everything seriously and worry				
32	I usually lack self-confidence				
33	I usually feel secure				
34	I avoid encountering difficult and stressful situations				
35	I usually feel sad				
36	I am usually happy with my life				
37	I am disturbed by random thoughts				
38	I take disappointments so seriously that I can never forget them				
39	I am a level-headed and determined person				
40	I am worried about the issues that have been on my mind lately				

ANNEX4 : Beck Depression Inventory (BDI)

1)	a. I don't feel sad<br>b. I feel sad<br>c. I'm sad all the time and I can't shake it<br>d. I'm so sad and unhappy I can't stand it
2)	a. I have no hope for the future<br>b. I have no hope for the future<br>c. I have nothing to expect from the future<br>d. There is no future for me and this situation will not change
3)	a. I don't see myself as a failure<br>b. I've had more failures than anyone else<br>c. When I look back, I see that I've had many failures<br>d. I see myself as a total failure as a person
4)	a. I can get as much satisfaction (pleasure) from everything as I used to<br>b. I can't get as much satisfaction from everything as I used to<br>c. I can't get real satisfaction from anything anymore<br>d.There is nothing that gives me satisfaction. Everything is so boring
5)	a. I don't feel guilty<br>b. I feel guilty sometimes<br>c. I feel guilty most of the time<br>d. I feel guilty all the time
6)	a. I don't feel like I'm being punished<br>b. I feel like I might be punished for some things<br>c. I feel like I'm going to be punished<br>d. I'm being punished for some things
7)	a. I'm not disappointed in myself<br>b. I'm disappointed in myself<br>c. I don't like myself at all<br>d. I hate myself
8)	a. I don't see myself as worse off than other people<br>b. I criticize myself for my weaknesses and mistakes<br>c. I always blame myself for my mistakes<br>d. I blame myself for everything bad that happens

9)	a. I don't have thoughts of killing myself<br>b. Sometimes I think of killing myself but I can't do it<br>c. I wish I could kill myself<br>d. If I get the chance I will kill myself
10)	a. I don't think I cry more than anyone else<br>b. I cry more now than I used to<br>c. I cry all the time now<br>d. I used to be able to cry. Now I can't cry even if I want to

11)	a. I am not more nervous or anxious than I used to be<br>b. I am a little more anxious than usual<br>c. I am nervous and anxious most of the time<br>d. I am nervous and anxious all the time now
12)	a. I haven't lost interest in other people<br>b. I'm less interested in people than I used to be<br>c. I've lost most of my interest in other people<br>d. I've lost most of my interest in other people
13)	a. I can make decisions as easily and comfortably as before<br>b. I postpone making decisions more than before<br>c. I have a lot more difficulty making decisions than before<br>d. I can't make decisions at all anymore
14)	a. I don't think I look worse than before<br>b. I feel like I've gotten older and lost my attractiveness and I feel sad<br>c. I feel like there are changes in my appearance that can no longer be changed and that make me look ugly<br>d. I think I'm very ugly
15)	a. I can work as well as I used to<br>b. I try harder than I used to to start a job<br>c. No matter what, I push myself hard to do it<br>d. I can't work at all anymore
16)	a. I can fall asleep as easily and comfortably as before<br>b. I can't fall asleep as easily and comfortably as before<br>c. I wake up an hour or two earlier than before and have difficulty falling asleep again<br>d. I wake up much earlier than before and can't fall asleep again
17)	a. I don't think I get tired any faster than I used to<br>b. I get tired quicker and easier than I used to<br>c. I get tired easily and quickly from almost everything these days<br>d. I'm too tired to do anything anymore
18)	a. My appetite is not much different than before<br>b. My appetite is not as good as before<br>c. My appetite is quite bad now<br>d. I have no appetite anymore
19)	a. I don't think I've lost/gained much weight lately<br>b. I've lost/gained more than two and a half kilos lately, even though I didn't want to<br>c. I've lost/gained more than five kilos lately<br>d. I've lost/gained more than seven and a half kilos lately
20)	a. My health doesn't worry me much<br>b. I've been having problems lately, such as pain, discomfort in the stomach, and constipation<br>c. These problems, such as pain and discomfort, worry me a lot<br>d. These kinds of problems worry me so much that I can't think of anything else

21) a. There is nothing that has caught my attention in my sexual life lately

b. I am less interested in sexual matters than I used to be

c. I am not very interested in sex these days

d. I have no interest in sex anymore

ANNEX5: Interpersonal Needs Questionnaire and Acquired Suicide Efficacy- Death Fearlessness Scale

Interpersonal Needs Questionnaire (INQ): First 10 questions

Acquired Suicidal Efficacy- Death Fearlessness Scale (ASE/DFS): Next 7 questions

		Strongly Disagree	Disagree	Sometimes Disagree	Undecided	Sometimes Agree	Agree	Kesinlikle Katılıyorum
1	These days, I think the people in my life would be better off without me.							
2	These days, I think that the people in my life would be happier without me.							
3	These days, I think that my death would be a relief for the people in my life.							
4	These days, I think that the people in my life want to get rid of me.							
5	These days, I think that I make things harder for the people in my life.							
6	These days, I feel like I belong to a family, a group, or a place.							
7	These days, I feel lucky to have many friends who care about me and support me.							
8	These days, I feel disconnected from other people.							
9	These days, I feel like a stranger in social situations.							
10	These days, I feel close to people.							
11	The fact that I will die one day doesn't affect me.							
12	I am very afraid of dying.							
13	It doesn't bother me when people talk about death.							
14	The fact that I will die worries me.							

15	Knowing that the end of life is death doesn't bother me.							
16	I am not afraid of dying.							
17	The pain of dying frightens me.							

ANNEX6: General Attitudes and Beliefs Scale Short Form (GABS-SF)

**If you completely agree**	5
**If you agree**	4
**If you somewhat agree**	3
**If you rarely agree**	2
**If you do not agree at all**	1

please mark the numbers.

1. Failing at things that are important to me is unbearable, and I can't stand failing.	① ② ③ ④ ⑤
2. I can't stand it when other people don't take me into account, and I can't stand being treated unfairly.	① ② ③ ④ ⑤
3. I can't stand being uncomfortable, stressed and tense, stress and tension are unbearable.	① ② ③ ④ ⑤
4. Even if I don't succeed at things that are important to me, I continue to feel valuable as an individual.	① ② ③ ④ ⑤
5. I can't stand being tense and stressed, I think being in extreme tension is unbearable.	① ② ③ ④ ⑤
6. It sucks when people close to me don't like me, it's terrible when they don't like me.	① ② ③ ④ ⑤
7. If the people close to me do not like me, it is because I am not a person worth liking.	① ② ③ ④ ⑤
8. When I am treated inconsiderately, it reminds me of how many evil and undesirable people there are in the world.	① ② ③ ④ ⑤
9. Even if I am rejected by someone I love, I can accept myself as I am and still continue to think that I have value as a person.	① ② ③ ④ ⑤
10. If I cannot perform the tasks that are important to me well, this is an indication that I am a worthless, useless person.	① ② ③ ④ ⑤
11. Underperforming on things that matter sucks, and those situations are scary to me.	① ② ③ ④ ⑤
12. I find it disastrous when people treat me disrespectfully.	① ② ③ ④ ⑤
13. When people reject me or don't like me, it's because I'm useless and worthless.	① ② ③ ④ ⑤
14. I can't stand being treated unfairly, and I think being treated unfairly is unbearable.	① ② ③ ④ ⑤
15. I think of those who wrong me as bad and worthless people.	① ② ③ ④ ⑤
16. I can't stand having difficulties in my life.	① ② ③ ④ ⑤
17. To be in difficulty is a terrible thing, and to be put in a difficult situation is a disaster.	① ② ③ ④ ⑤
18. It is unbearable not to be able to do important tasks well, and I cannot stand making mistakes in these tasks.	① ② ③ ④ ⑤
19. It is important for people to treat me fairly in general, but I am also aware that there is no obligation for me to be treated fairly.	① ② ③ ④ ⑤
20. It would be a terrible thing if I could not perform successfully in important tasks.	① ② ③ ④ ⑤

21. It is unbearable to be disrespected by people. I cannot stand disrespect.	① ② ③ ④ ⑤
22. If important people do not like me, it is a sign that I am a worthless person.	① ② ③ ④ ⑤
23. I have to be loved and accepted by the people I want to love me, and if they don't I can't accept that.	① ② ③ ④ ⑤
24. I want the people I love to love me and accept me for who I am, but I am also aware that if they don't, they don't have to love me just because I want them to.	① ② ③ ④ ⑤
25. It is unbearable when the people I want to love me disapprove or reject me and do not like me.	① ② ③ ④ ⑤
26. If people treat me disrespectfully, that's a sign of how bad people they really are.	① ② ③ ④ ⑤
